# Multicolor bioimaging with biosynthetic zinc nanoparticles and their application in tumor detection

**DOI:** 10.1038/srep45313

**Published:** 2017-03-27

**Authors:** Yanjun Kang, Yi-Zhou Wu, Xianyun Hu, Xueqin Xu, Jie Sun, Rong Geng, Tongxing Huang, Xiaohang Liu, Yichen Ma, Ying Chen, Quan Wan, Xiaobang Qi, Gen Zhang, Xiaohui Zhao, Xin Zeng

**Affiliations:** 1 Wuxi School of Medicine, Jiangnan University, 214122, China; 2 Department of Cell Biology, School of Basic Medical Sciences, Nanjing Medical University, 210029, China; 3 Department of Biochemistry, Qiannan Medical College for Nationalities, Duyun 558000, China; 4 The Center for Hygienic Analysis and Detection, School of Public Health, Nanjing Medical University, Nanjing 211166, China; 5 Key Laboratory of Tibetan Medicine Research, Northwest Institute of Plateau Biology, Chinese Academy of Sciences, Xining 810001, China; 6 Nanjing Maternity and Child Health Medical Institute, Obstetrics and Gynecology Hospital Affiliated to Nanjing Medical University, 210004, Nanjing, China

## Abstract

Because they generate excellent images, nanoparticles (NPs), especially biosynthesized NPs, provide a new solution for tumor imaging. In this research, we unveil a novel type of biosynthesized NPs featuring multicolor fluorescence. These NPs exhibit little cytotoxicity to cells. The explored NPs, designated Zn-ZFP-GST NPs (Zinc NPs in abbreviation), are generated from leukemia cells treated with a Zn^2+^ solution, while zinc-finger protein and glutathione S-transferase (GST) were also identified in the Zinc NPs. Under near-UV illumination, the Zinc NPs simultaneously emit green, yellow, and red fluorescence. In addition, the intensity of the fluorescence increases with the existence of sulfides. Besides, the NPs are encapsulated by microvesicles (MVs) shed from the plasma membrane. As observed in whole-body research of nude mice, the NP-MVs migrate via blood circulation and are distinguished by their fluorescent signals. Furthermore, the folic acid (FA) & AVR2 (human VEGF antibody)-coated NP-MVs are exploited to target the tumor location, and the feasibility of this approach has been confirmed empirically. The Zinc NPs shed light on an alternative solution to tumor detection.

Magnetic resonance imaging (MRI), positron emission tomography (PET) and X-ray computed tomography (CT) are the most-used imaging technologies for the detection of cancer to date. However, these technologies are still ineffective in early-stage cancer or tumor metastasis diagnosis^1^,^2^,^3^. In recent years, with the advance of nanotechnology, nanomaterials have been employed as new luminescent agents for biological imaging^4^,^5^,^6^,^7^,^8^,^9^. Luminescent nanoparticles (NPs) exhibit unique size, optical, and structural features. Briefly, luminescent NPs possess higher levels of brightness, photostability, and biocompatibility than other fluorescent organic dyes^10^,^11^,^12^. In addition, by virtue of their optical and size properties, these NPs demonstrate their great superiority in tumor imaging and tracing^11^,^13^,^14^. To date, a wide range of NPs have been developed for tumor diagnosis. Of the luminescent NPs, many types of quantum dots (QDs), such as CdSe, ZnS and other multiple material, doped QDs, are the most common and have been well described^15^,^16^,^17^. In addition, gold NPs and fluorophore-doped silica NPs have also been a frequent focus of research^18^.

For economic reasons, large quantities of NPs of variable sizes are traditionally synthesized by chemical methods^19^. However, chemically synthesized NPs possess patent flaws. The toxic heavy metals contained in the NPs may be harmful to the cells or organism^20^,^21^. To conquer this problem, many types of modified NPs have been developed by conjugating biocompatible materials, such as polyethylene glycol, a silica shell, or synthetic peptides. Otherwise, despite their complicated preparation process, the optical property of biocompatible materials is compromised^21^.

Contrarily, biosynthesized NPs are more biocompatible because of their environmentally friendly synthetic composition, which provides a promising solution for further application due to their facile and economically advantageous features. To date, mostly biological entities, such as mammalian cells, bacteria, and other organisms, have been exploited as the “factory” for metallic NP production. Several studies have exploited novel ways to synthesize the NPs biologically, at either the organism or cell level^18^,^22^,^23^,^24^,^25^.

A pioneering work revealed nanomaterial biosynthesis using tissues near the earthworm gut; the nanomaterials were subsequently coated with polyethylene glycol and were available for imaging of macrophage cells^24^. However, in addition to requiring surface modification, this synthetic procedure is obviously time consuming. In another study, gold nanoclusters were developed using cancer cells. This method has the advantage of a large amount of nanomaterial production due to rapid cell division^18^. It has been widely reported that the cells of many organisms are preferred for biosynthesizing nanoparticles with metal ions (e.g., Au^+^, Ag^+^ and Zn^2+^) due to their cost-effective and nontoxic properties^26^.

In this paper, we develop and characterize a novel type of microvesicle (MV)-encapsulated zinc NPs in leukemia cancer cells. These NPs simultaneously emit green, yellow, and red fluorescence signals, impose little cell toxicity and can be readily applied for *in vivo* imaging. Targeted tumor detection can be performed with antibodies attached to the MV surface, affording fluorescence images at different wavelengths and avoiding background interference by the multiple color fluorescence.

## Results

### Biosynthesis of Zinc NPs encapsulated by microvesicles in cancer cells

**Figure 1:**
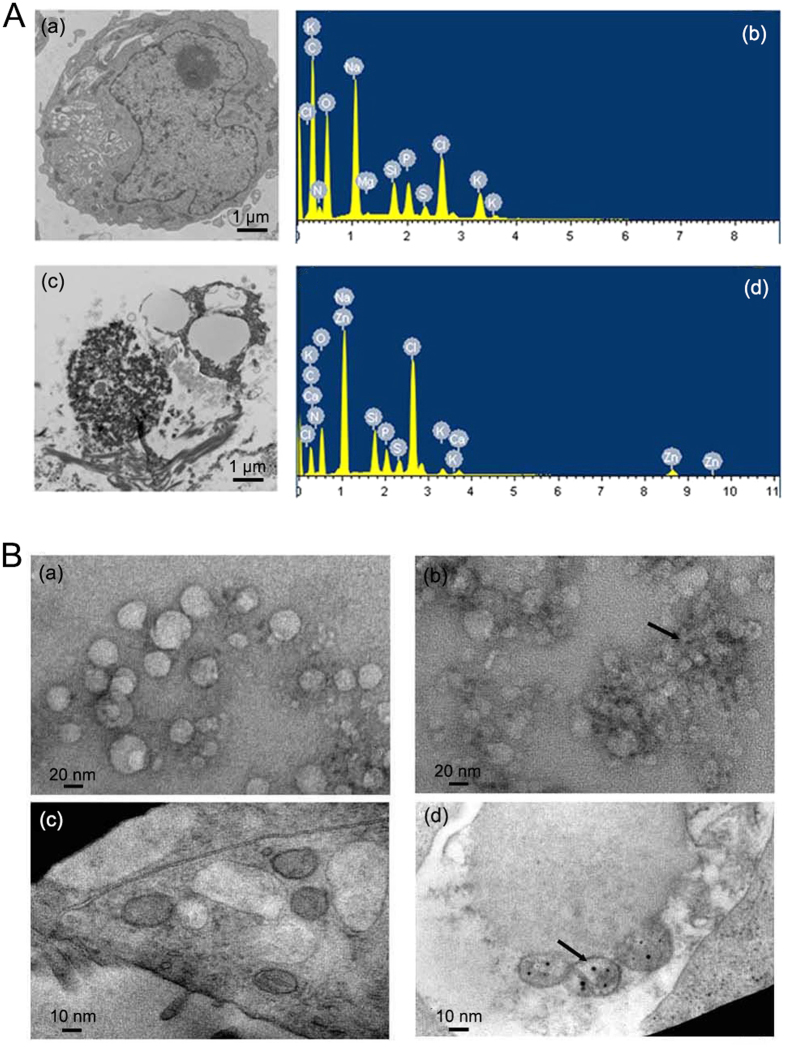
(**A**) Morphological and compositional characterization of Zn^2+^-treated tumor cells: (a) and (c) show TEM images at the sub-cellular level of control and Zn^2+^-treated cells, respectively; (b) and (d) show the corresponding results of the EDS analysis. (**B**) TEM images of MVs from (a) Zn^2+^-treated KA cells; (b) Zn^2+^-treated KA cells under ultrasound treatment ( → ); (c) KA cells without treatment; (d) Zn^2+^-treated KA cells under ultrasound treatment (→).

The functionalized zinc-derived NPs were synthesized and characterized by transmission electron microscopy (TEM) imaging. In the case of KA cells incubated with Zn^2+^ solutions, the TEM image (Fig. 1A c) displayed typical microstructure changes in tumor cells compared with those in untreated cells (Fig. 1A a). Energy dispersive X-ray spectroscopy (EDS) observation further indicated that the calculated atom content of Zn in cancer cells changed significantly after incubation with Zn^2+^ solutions. The microstructurally altered cells clearly showed MVs with diameters of 30–50 nm (Fig. 1B a). Using facile ultrasound treatment, the disrupted cells effectively presented the MV-loaded NP complexes under TEM at different magnification scales. The TEM assay verified that, compared with the control (Fig. 1B c), the MVs derived from Zn^2+^-treated KA cells could encapsulate NPs (Fig. 1B b and B d, arrow indicator). The particle size varied between approximately 4 and 6 nm (Fig. S1). The nanoparticles were generated approximately two hours after the zinc solution was added. The number of NPs increased with the duration of incubation and reached a peak in the 48 h treatment (Fig. S2). EDS results (Figs S3 and 4) confirmed the content of Zn in the MVs in cancer cells. In addition, the surface charge (zeta potential) of the Zinc NP-MVs is −17.1 mV, which indicates that the NP-MVs are negatively charged when dispersed in water. The results of MTT assays demonstrated that the Zinc NPs did not show apparent cytotoxicity even at high concentrations (Fig. S5).

### The multiple fluorescences of Zinc NP-MVs in cancer cells

**Figure 2:**
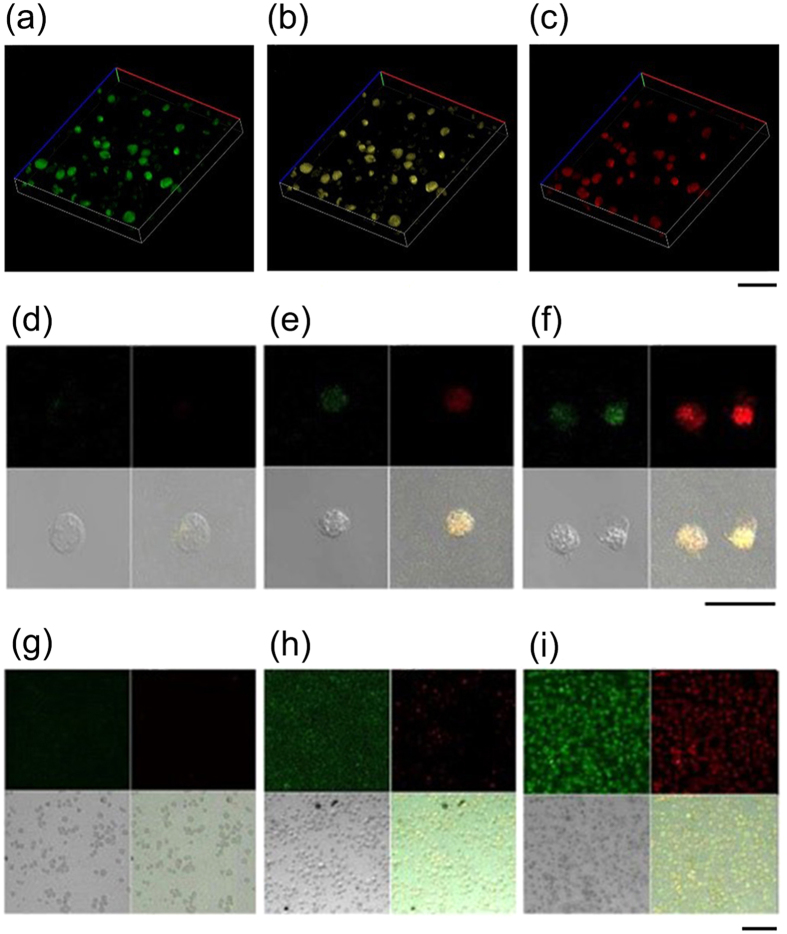
Fluorescent image of the 3D reconstruction of KA cells treated with 10 mmol/L Zn^2+^ solutions after 24 h incubation for green (**a**), yellow (**b**), and red (**c**) intracellular fluorescence; scale bar: 100 μm; laser confocal fluorescence micrographs (400-fold magnification) of KA cells incubated with 10 mM Zn^2+^ solutions for 0 h incubation (**d**), 12 h incubation (**e**) and 48 h incubation (**f**); scale bar: 50 μm; laser confocal fluorescence micrographs (100-fold magnification) of KA cells incubated with 10 mM Zn^2+^ solutions for 0 h incubation (**g**), 12 h incubation (**h**) and 48 h incubation (**i**); scale bar: 100 μm.

**Figure 3:**
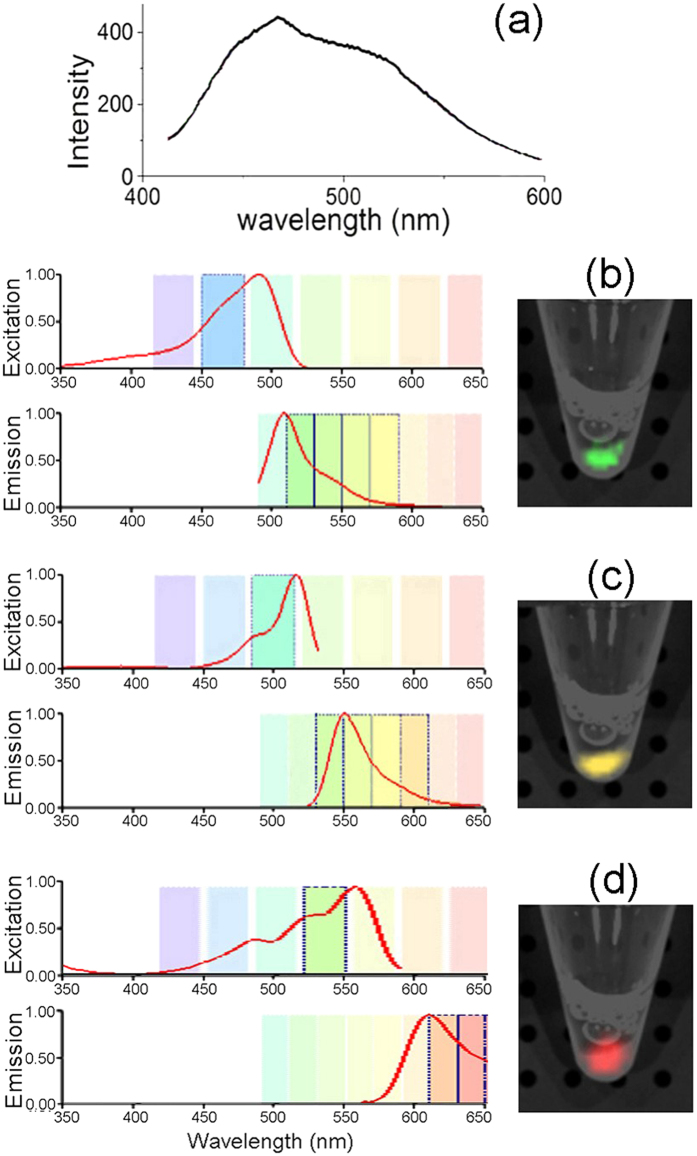
Optical properties of NP-MVs. (**a**) The visible absorption spectrum of the NP-MVs obtained from KA cells pre-exposed to 10 mM Zn^2+^ for 12 h. Excitation and emission spectra of MV-NPs corresponding to green fluorescence at 520 nm (**b**), yellow fluorescence at 560 nm (**c**) and red fluorescence at 620 nm (**d**). On the right of panels (**b**–**d**) are images of the NP-MVs cast in phosphate buffer with excitation wavelengths set at 480, 520, and 560 nm.

**Figure 4:**
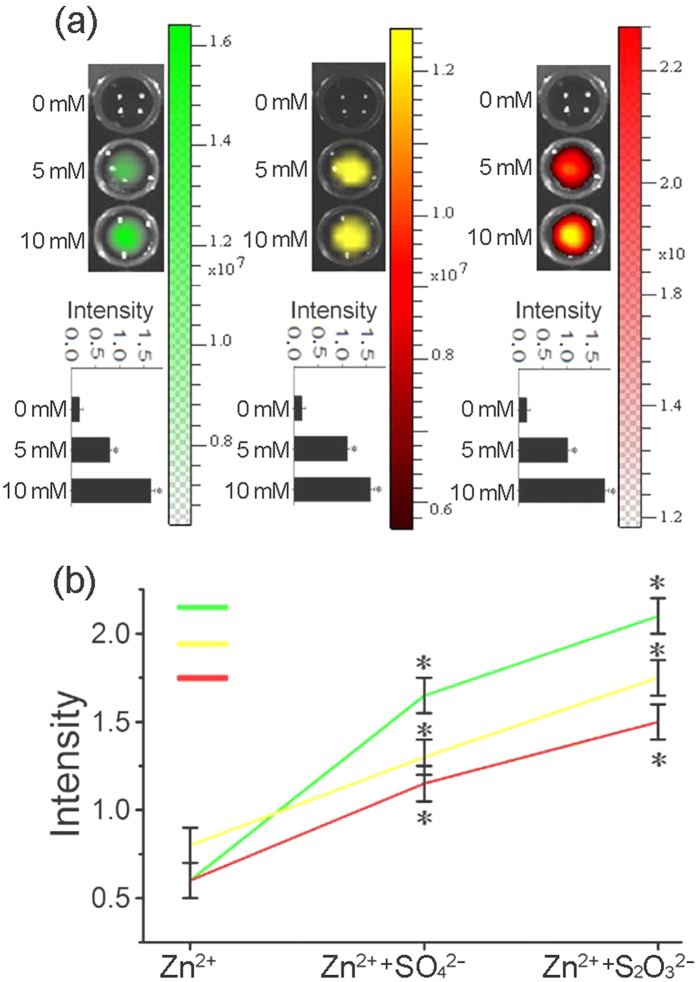
Dependence of NP-MV quantities on Zn^2+^- and S-containing ions in the cell media. (**a**) Fluorescence images of KA cells with pre-exposure to 0, 5 and 10 mM Zn^2+^ solutions for 12 h. The green, yellow, and red fluorescence were obtained with excitation wavelengths set at 480, 520, and 560 nm. (**b**) Plots of green, yellow, and red fluorescence intensities against the ions to which KA cells were exposed.

After treatment by Zn^2+^, the imaging properties of KA cells were examined under a fluorescent microscope. The 3D-reconstructed fluorescent images give a clear overall view of the green, yellow and red fluorescence, which demonstrated that the three-color fluorescence appears higher in intracellular distribution (Fig. 2a–c). Furthermore, the magnifying scope illustrated the three color features at 400-fold and 100-fold magnification (Fig. 2d, 2e, 2f, 2g, 2h, 2i). We can see in the magnification imaging that the fluorescence intensity increases with a longer zinc treatment. In addition, zinc-treated acute myeloid leukemia (AML) cells shared the same fluorescent features (Fig. S6). The absorption range of the zinc-treated cells varied between 400 and 600 nm, of which the optimum excitation spectra at 480, 520 and 560 nm were applied to excite green, yellow, and red fluorescence, respectively (Fig. 3b,c,d). In agreement with this approach, the biosynthesized Zinc NPs in KA cells displayed three accordingly typical emission spectra peaking at approximately 520, 540 and 580 nm. The various fluorescence intensities emitted by the Zinc NPs increased with higher Zn^2+^ concentration in the KA cells (Fig. 4a). Furthermore, the S concentration can influence the fluorescence intensity, indicating the S may contribute the chemical characteristics of the NPs (Fig. 4b). Na_2_S and Na_2_SO_4_ shared a similar combination property linked with fluorescent imaging for the biosynthesized Zinc NPs, while Na_2_S_2_O_3_ showed stronger fluorescence.

### Self-imaging and migrating capacity of zinc-derived NPs *in vivo*

**Figure 5:**
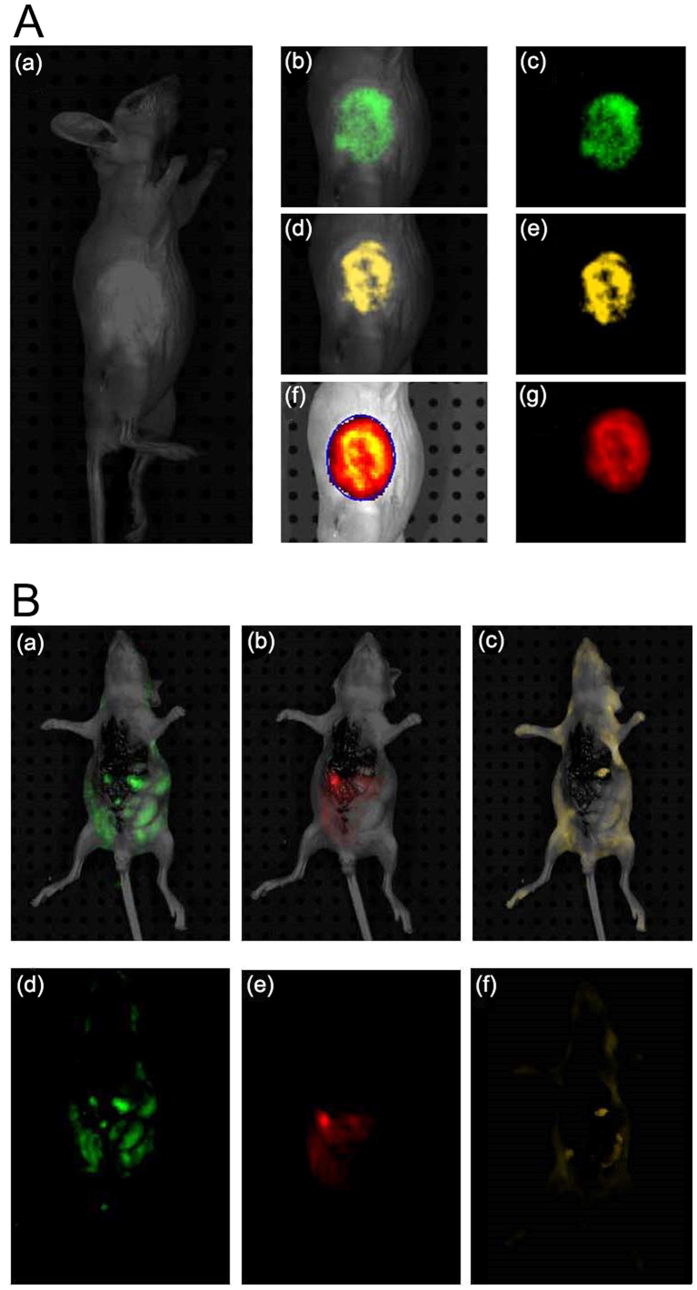
*In vivo* imaging with NP-MVs. (**A**) *In vivo* tumor images of KA tumor-bearing mice 24 h after being treated with 10 mM Zn^2+^ solutions: (bc, de, fg) for green, yellow, and red fluorescence distribution in the tumor location, respectively; bright field image (a). (**B**) Fluorescence images of an anesthetized nude mouse that had been subjected to portal vein injection of NP-MVs with excitation wavelengths set at 480 (a), 520 (b), and 560 nm (c).

In this study, Zinc NP-MVs were mainly expected to be applied for tumor imaging by functional biomolecules. *In vivo* imaging results showed significant green (Fig. 5A b,c), yellow (Fig. 5A d,e) and red (Fig. 5A f,g) fluorescence in the animal body after treatment with zinc salts. The three-color fluorescence was captured in the spectra excited at 480, 520 and 560 nm.

To investigate the migratory capacity of the Zinc NP-MVs around the blood vessels in the animal body, the produced NP-MVs were injected into the body of nude mouse via the hepatic portal vein to detect the self-imaging signals. The results revealed that strong and specific green (Fig. 5B a,d), yellow (c,f) and red (b,e) fluorescence signals at 520, 540 and 580 nm emission wavelengths respectively, were detected in the whole-body imaging.

### Tumor-targeted imaging of the Zinc NP-MVs *in vivo*

To validate the feasibility of the tumor targeting feature of the NP-MVs, an experiment on blood cell carcinoma tumor-bearing nude mice was conducted. Because the tumor-specific antibodies FA and AVR2 are positively charged, the MVs can readily absorb the antibodies.

**Figure 6:**
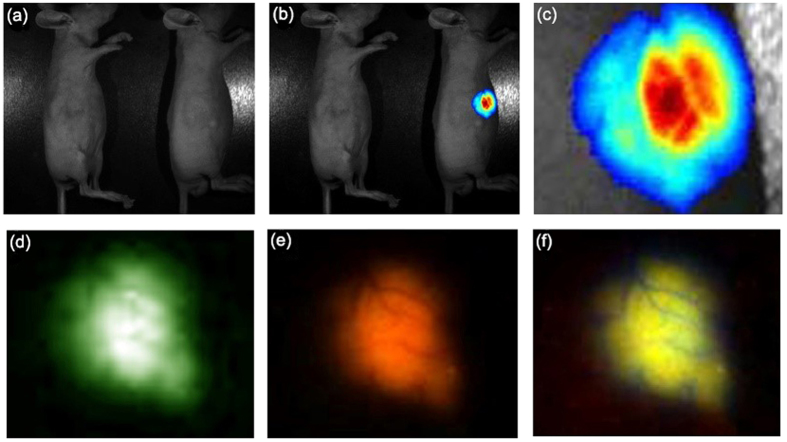
*In vivo* imaging with NP-MVs-FA/AVR2. Images (**b**) were obtained under UVillumination from mice containing NP-MVs (left) and AVR2/FA-coated NP-MVs (right). Panels (**d**–**f**) show enlarged images of the tumor in the mouse injected with AVR2/FA-coated NP-MVs; bright field image (**a**).

The results clearly demonstrated that the bright fluorescence was located around the tumor, and the blood vessels were also distinguishable (Fig. 6). In accordance with the previous results, the tumor distribution was displayed by the three-color fluorescence. These results contrasted with those obtained for the control, which was injected with only NP-MVs (Fig. 6b). Thus, the FA/AVR2-coated NP-MVs accumulated at the location of the tumor.

### Compositional characteristics of biosynthetic Zinc NPs

**Figure 7:**
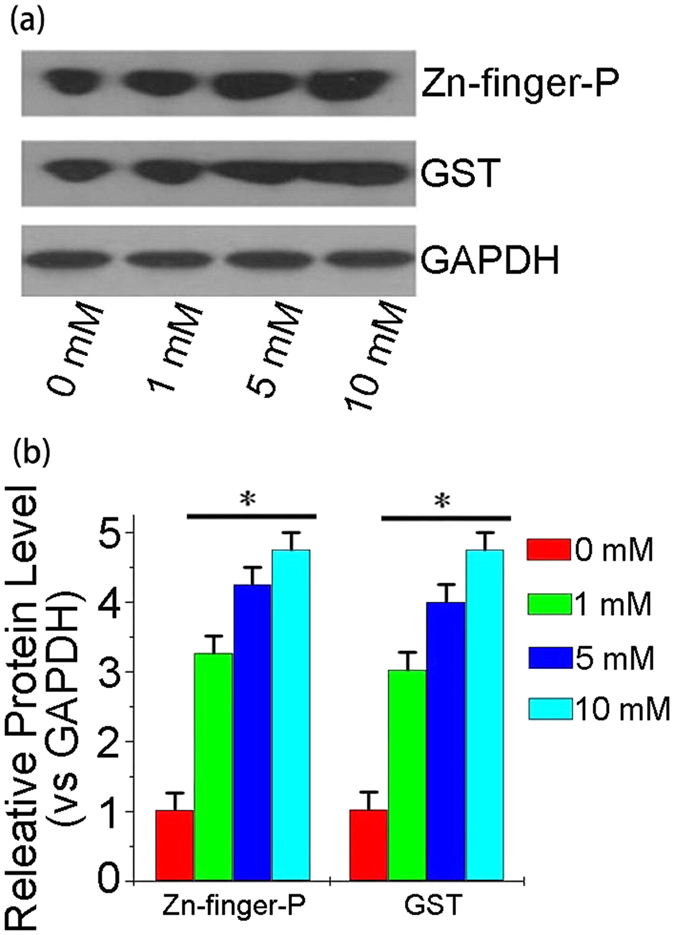
Composition of the Zinc NPs. (**a**) Western blot analysis of Zn-finger proteins and GSH-transfer-s-protein in lysates of KA cells exposed to different Zn^2+^ concentrations. GADPH was used as a loading control. The western blot images were cropped to appropriate sizes. (**b**) A bar graph showing the relative concentrations of Zn-finger proteins and GST vs. GADPH under a gradient concentration of Zn^2+^ treatment.

To gain insight into the mechanism underlying the biosynthesis of MV-NPs in KA cells, we used mRNA chips to gauge the expression levels of messenger RNAs (Supplementary Table S1). Two mRNAs, corresponding to Zn-finger protein (ZFP) and glutathione-transfer-S-protein (GST), were significantly elevated. Indeed, from the western blot analysis (Fig. 7), it is evident that the concentrations of ZFP and GST are both up-regulated in Zn^2+^-treated KA cells.

## Discussion

In addition to being an essential trace element in the human body, zinc is also used to synthesize metallic NPs and has become increasingly prevalent in recent years^27^. For example, zinc oxide (ZnO) nanoparticles display various novel features and potential applications. In addition, in many types of multiple material, doped QDs, ZnS serves as an essential substrate^28^,^29^. Most of these NPs are produced via chemical procedures, including some modified methods^30^. While these chemically modified NPs are considered compatible with the organism, the quality of their optical properties is reduced to some extent. Due to their relatively low toxicity, biosynthesized NPs have attracted increasing interest in recent years. In a previous study, we reported the green fluorescence of gold nanoclusters biosynthesized *in situ* inside HepG2 and K562 cancer cells treated by HAuCl_4_, demonstrating that gold nanoclusters can be used successfully for *in vivo* bio-imaging of relevant live tumor cells^18^. Now, through our study on biosynthesized zinc-derived NPs, we find that the biosynthesized zinc NPs can emit multiple-color fluorescence and that sulfur can enhance the fluorescence intensity. Because ZFP and GST have been identified as essential components of the NPs, the NPs are designated Zn-ZFP-GST NPs, or “Zinc NPs”. The uncovered novel Zinc NPs possess fluorescent wavelengths from 500 nm to 600 nm from the green to red fluorescence. The three-color fluorescence emissions at different wavelengths can circumvent interference from autofluorescence or endogenous or exogenous fluorescent molecules in the living body.

NPs featuring multiple-color fluorescence have been reported frequently^31^,^32^. Generally, the earlier-reported NPs emitting multiple-color fluorescence were chemically modified by a bonding ligand, which may reduce biocompatibility. In contrast, the Zinc NPs in this research are biosynthesized in a green fashion. Notably, the secreted Zinc-ZFP-GST NPs from zinc-treated cells are naturally enclosed in MVs. MVs are fragments of the plasma membrane-derived liposome that are shed from almost all cell types under normal or pathological conditions^33^,^34^. To date, MVs have been mainly studied by biological methods, which remains challenging due to their comparatively small size of 50–100 nm. For a long time, the liposome was used to enclose QDs to reduce their cytotoxicity and enhance their biocompatibility^35^. Otherwise, the targeting ability of liposomes can be ameliorated by an antibody combination. Because the MVs can be regarded as endogenous cell organelles, the resulting Zinc NPs are naturally innocuous to cells. In addition, an MTT assay confirmed that Zinc NPs are not cytotoxic, even at high doses, and can therefore be used as a new agent for *in vivo* imaging. To our knowledge, this is the first report of biosynthesized NPs encapsulated by microvesicles.

Because Zn-finger protein and GST are known to bind to a variety of cellular toxins for detoxification purposes^36^,^37^, the up-regulation of the two proteins is likely related to the sequestration and transport of Zn^2+^. Na_2_S_2_O_3_ shows stronger fluorescence than Na_2_S and Na_2_SO_4_, which strongly suggests that S plays an important role in the NP generation phases. Therefore, we believe that sulfur promotes the formation of a mercapto group (-SH) of the ZFP or GST, which further benefits the binding with Zn^2+^. Accordingly, we predict that the Zn-ZFP-GST complex generated through the oxidation reaction possesses a unique excitation spectrum. Clearly, deciphering the precise luminescence mechanism of the zinc NPs requires additional research.

It is generally believed that NP imaging has the potential to lead to a better understanding and management of cancer-targeted therapies^11^,^38^. Additionally, imaging techniques must be convenient to apply to optimize targeted strategies to different tumor entities before entering clinical application. Our research revealed that the subcutaneous injection of a millimolar Zn^2+^ solution near xenograft tumors of the nude mouse led to the efficient biosynthesis of triply-fluorescent NPs, allowing specific fluorescent self-bio-marking of the tumors. To specifically target tumors, we attached FA/AVR2 to the MV-NP surfaces. The two tumor antibodies are positively charged and can be readily adsorbed onto the negatively charged MV-NPs. Figure 4e clearly shows that the AVR2-coated MV-NPs accumulate at the tumor surface. This result is in contrast to that shown in Fig. 4d, which shows an image recorded from a mouse injected with only MV-NPs. As Fig. 6 shows, the target tumors can be distinguished clearly by the fluorescence imaging of the NPs after the Zinc NP-MVs-FA/AVR2 are introduced into nude mice via intravenous injection.

This study also shed new light on the diagnosis of tumor metastasis. Cancer progression and metastasis can result in the accumulation and concentration of fluorescence that labels the cells at metastatic regions. Hence, metastasis can be observed through fluorescence imaging^9^. Injection of cancer cells into the tail vein of mice can stably express green fluorescent protein (GFP), and it is feasible to visualize single tumor cells in blood vessels^39^,^40^. In our research, after the injection of Zinc NPs through the hepatic portal vein, strong and specific green, yellow and red fluorescent signals were distinct in different internal organ autopsy images. In addition, the migration of the NP-MVs via blood circulation indicates that the MV-encapsulated NPs are benign to the animal body. Because the three-color-fluorescence Zinc NPs can migrate through blood vessels in the animal body, we can anticipate that this novel material has the potential for tracing metastasis.

In conclusion, our research has revealed a novel method for the biosynthesis and MV encapsulation of Zinc NPs in a cost- and time-effective manner. The NPs simultaneously emit green, yellow, and red fluorescence under near-UV illumination and exhibit little cytotoxicity to the organism. Consequently, these NPs are potentially amenable for *in vivo* imaging and multi-color tumor detection.

## Methods

### Biosynthesis and imaging characterization of Zinc NPs *in vitro*

The adriamycin-resistant leukemia cell lines K562/A02 (KA) were maintained in RPMI-1640 medium containing 10% fetal calf serum (FCS), 100 U/mL penicillin, 1 g/mL adriamycin and 100 g/mL streptomycin at 37 °C with 5% CO_2_. A sterile coverslip was put on the bottom of the cell culture dish for the latter assay. Zinc chloride (ZnCl_2_) dissolved in distilled water was added into the cell media (containing 10^7^ KA cells) to a predetermined concentration (10 mM, 5 mM, and 0 mM for control), and the cells were incubated overnight. To isolate cells, cell media were centrifuged for 5 min at 900 × g, and to remove remaining debris, another centrifugation step was performed for 1 h at 10,000 × g. The morphologies of the cells were characterized by TEM (JEM-2100, Japan) and EDS. To examine the correlation between the duration and the quantity of NPs, the number of NPs were calculated using the Zetasizer Nano (Malvern Instrument, UK) at different zinc treatment time points (1, 2, 4, 8, 12, 24, 48 and 72 h).

The KA cells (5 × 10^5^/well) on coverslips were examined under a fluorescent light microscope and then centrifuged for 5 min (400 g) for the examination of small animal whole-body optical imaging. 3D reconstructions of (5 × 10^5^/well) Zn^2+^-treated KA cells were established to reveal the intracellular distributions of fluorescence signals. After treatment with Zn^2+^, various sulfides with the same concentration as Zn^2+^ were separately added to the cells for 12 h. Finally, the KA cells were centrifuged for 5 min (400 g) and examined under small animal whole-body optical imaging and other experiments.

The cytotoxic effects of the zinc NPs on the KA cell line were examined by an MTT assay. The cell growth inhibition rate was monitored after a 36 h treatment with a concentration gradient of Zn^2+^.

### Isolation and characterization of biosynthetic Zinc NP-MVs *in vitro*

The generated Zinc NPs encapsulated by MVs were isolated from cell culture supernatants of KA cells. To remove residual cells, the media were first centrifuged for 5 min at 900 × g followed by centrifugation for 1 h at 10,000 × g to remove the last remaining debris. Afterwards, the supernatants were filtered through 0.2 μm pore filters to remove particles bigger than 200 nm. To concentrate the MVs, the filtrate was passed through a Vivacell 100 Filter (Sartorius AG, Goettingen, Germany) during 30 min centrifugation at 4 °C and 400 × g. Then, 10 mL of the concentrated supernatant were passed through a Sepharose CL-2B column (1.5 cm × 45 cm; GE Healthcare, Munich, Germany), and 1 mL fractions were pipetted. The filtrate was then lyophilized using a freeze-dryer. In parallel, the obtained precipitates were also resuspended in 150 μL of phosphate buffered saline (PBS) for further colloid chemical analyses. Finally, the separated Zinc MV-NPs were also characterized by TEM, EDS and fluorescence spectroscopy (Shimadzu UV3150, Japan).

### Fluorescence imaging and migration of biosynthetic Zinc NP-MVs *in vivo*

To explore the imaging feasibility of the Zinc NPs *in vivo*, small animal entire-body optical imaging was performed. All animals were housed in the Animal Care Facility of Nanjing Medical University under standard approved laboratory conditions. Animal procedures were performed in accordance with protocols approved by the Animal Ethical and Welfare Committee of NJMU (Application No. 10799 & Approval No. IACUC-1601234). Diethyl ether was used for anesthesia followed by cervical dislocation.

Nude mice were utilized in the *in vivo* imaging test. First, 20 μl of 1 mol/L Zn^2+^ was injected into the abdomen of animals via subcutaneous injection. After 24 h, the mice were anesthetized by inhalation of a mixture of oxygen with 5% isoflurane and then placed supine on the table so that the detector could be positioned in the tumor region of the animal. A small animal model *in vivo* imaging instrument (Carestream Multispectral, Canada) was used in the fluorescence detection.

To evaluate the migrating capacity of the produced NPs in the animal body, the KA cells bearing Zinc NP-MVs through the nude mice were traced by the fluorescence signal. The cells (≈10^6^) were injected into the animal body via the hepatic portal vein. After 24 h, the mice were anesthetized by isoflurane. Before the fluorescence detection, the abdomen of the nude mice was opened to observe the fluorescence with higher intensity.

### Biosynthesis and tumor locating feasibility of tumor-specific Zinc NP-MVs

To target the tumor location in the animal body, the monoclonal tumor-specific antibodies AVR2 and FA were applied to the Zinc NP-MVs. Briefly, the solution, which was composed of AVR2/FA/Zinc NP-MVs in a 1:1:10 proportion (mass ratio) reacted overnight to allow complete absorption. The resulting nanocomposite was then centrifuged at 40,000 rpm for 20 min. To evaluate the status of the antibody binding to the NP complex, the antibody remaining in the supernatant was quantified using a protein assay kit (Thermo Scientific, USA).

For bio-imaging the xenograft tumor, the mixture was injected into the blood of nude mice. The animals were anesthetized by inhalation of a mixture of oxygen with 5% isoflurane 24 h after injection. An excitation spectrum with different wavelengths was used for the tumor detection imaging. The atom content of the tumor cells was detected in deparaffinized tumor tissue sections using EDS.

The nude mice bearing xenograft tumors were established first to pursue the bio-imaging and locating feasibilities of the Zinc NPs-MV-FA/Zinc NPs-MV-AVR2 mixture in the living body. The mice were treated with subcutaneous injections of K562 cells (n ≈ 10^7^), and the weight of the solid tumor could not exceed 10% of the entire mouse body, which took approximately 1 week. Afterwards, 100 μl of the mixture was injected into the mouse tumor xenograft model through the caudal vein. After 30 min, the mice were anesthetized by inhalation of a mixture of oxygen and 5% isoflurane, and the three-color fluorescent signal was examined.

### Composition identification of biosynthetic Zinc NPs

After a 12 h treatment with Zn^2+^ (10 mM), changes in the transcriptional products of KA cells (1 × 10^7^ cells/well) were analyzed by Human Transcriptome Array 2.0 (Affymetrix, USA), which was conducted by the commercial service of Capitalbio Corporation, China. Based on the mRNA chip results, the targeted protein was further confirmed by an immunological method. The treated cell lysates were then subjected to sodium dodecyl sulfate polyacrylamide gel electrophoresis (SDS-PAGE)/western blot analysis with specific primary antibodies. The results were visualized by enhanced chemiluminescence (ECL, Thermo Scientific, USA). The western blot result was scanned using Quantity One software (Bio-Rad, USA).

## Additional Information

**How to cite this article:** Kang, Y. *et al*. Multicolor bioimaging with biosynthetic zinc nanoparticles and their application in tumor detection. *Sci. Rep.* **7**, 45313; doi: 10.1038/srep45313 (2017).

**Publisher's note:** Springer Nature remains neutral with regard to jurisdictional claims in published maps and institutional affiliations.

## Supplementary Material

Supplementary Dataset 1
