# Supplementary material for: Multicolor bioimaging with biosynthetic zinc nanoparticles and their application in tumor detection
Source: Sci Rep. 2017 Mar 27;7:45313. doi: 10.1038/srep45313 (PMC5366897; doi:10.1038/srep45313)
Supplement: Supplementary Dataset 1 [file srep45313-s1.doc]

**Supporting information**

Multicolor bioimaging with biosynthetic zinc nanoparticles and their application in tumor detection

Yanjun Kang^1†^, Yi-zhou Wu^2†^, Xianyun Hu^4†^, Xueqin Xu^2^, Jie Sun^5^, Rong Geng^2^, Tongxing Huang^2^, Xiaohang Liu^2^, Yichen Ma^2^, Ying Chen^2^, Quan Wan^2^, Xiaobang Qi^2^, Gen Zhang^2*^,

Xiaohui Zhao^6*^ and Xin Zeng^3*^

1 Wuxi School of Medicine, Jiangnan University, 214122, China

2 Department of Cell Biology, School of Basic Medical Sciences, Nanjing Medical University, 210029, China

3 Nanjing Maternity and Child Health Medical Institute, Obstetrics and Gynecology Hospital Affiliated to Nanjing Medical University, 210004 Nanjing, China

4 Department of Biochemistry, Qiannan Medical College for Nationalities, Duyun 558000, China

5 The Center for Hygienic Analysis and Detection, School of Public Health, Nanjing Medical University, Nanjing 211166, China

6 Chinese Acad Sci, Northwest Plateau Inst Biol, Key Lab Tibetan Med Res, Xining 810001, China

†These authors contributed equally to this work.

∗ Corresponding authors:

Gen Zhang: zhanggen@njmu.edu.cn

Xiaohui Zhao: xhzhao@nwipb.cas.cn

Xin Zeng: august555482@126.com


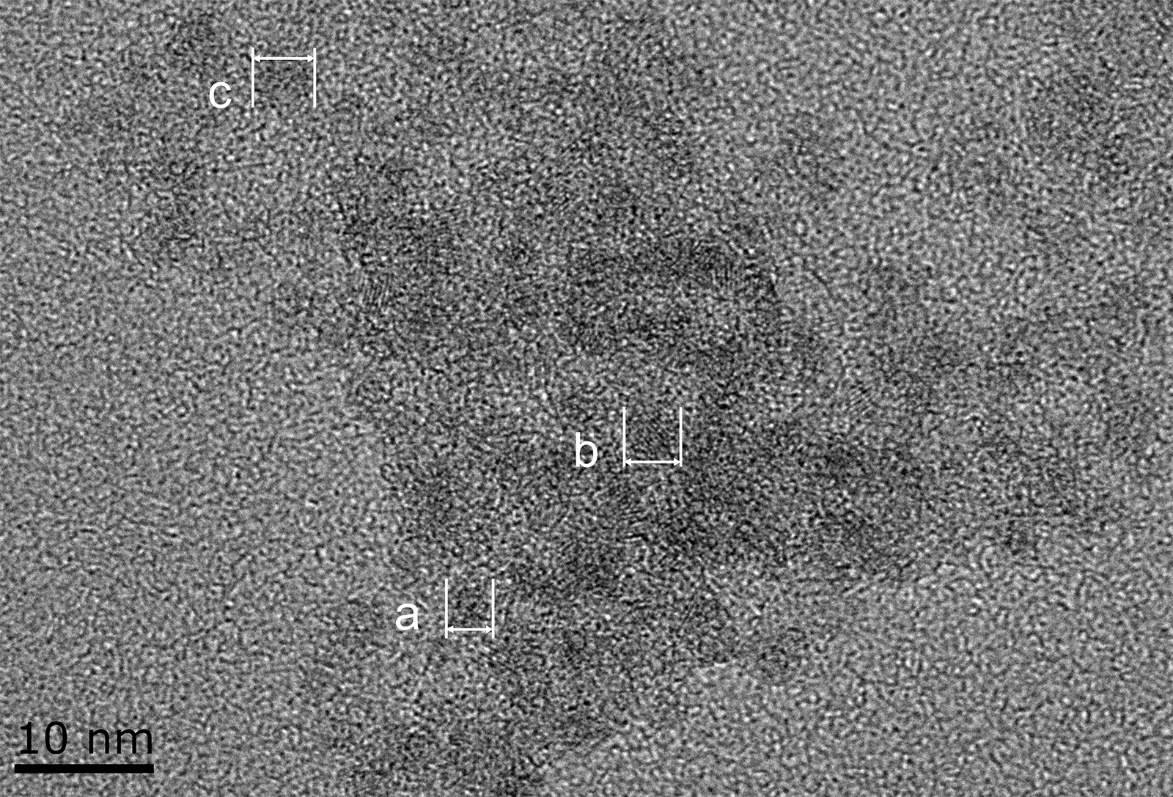


Figure S1.

TEM image of the zinc NPs. The labels a, b and c indicates the NPs with approximate diameter of 4, 5 and 6 nm respectively.


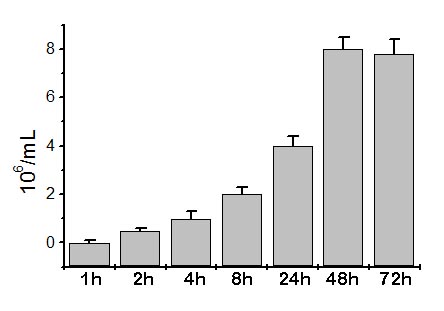


Figure S2.

The numbers of the zinc NPs with the duration of the zinc treatment. The numbers of the NPs were calculated using the Zetasizer Nano (Malvern Instrument).

**
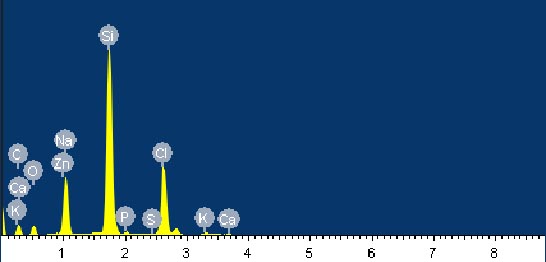
**

Figure S3.

EDS analysis from Zinc-ZnFP-GTP NPs distributed in MVs from KA cells (TEM images of MVs released from KA cells treated with Zn^2+^ in Fig. 1. B, b).


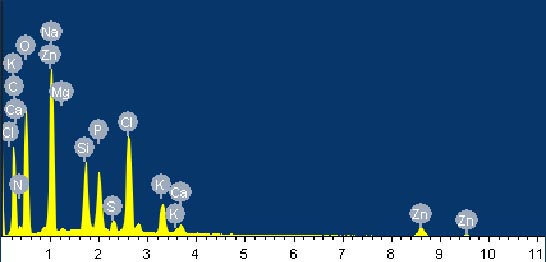


Figure S4.

EDS analysis from Zinc-ZnFP-GTP NPs distributed in MVs in KA cell slices (TEM images of MVs in KA cells treated with Zn^2+^ in Fig. 1. B, d).


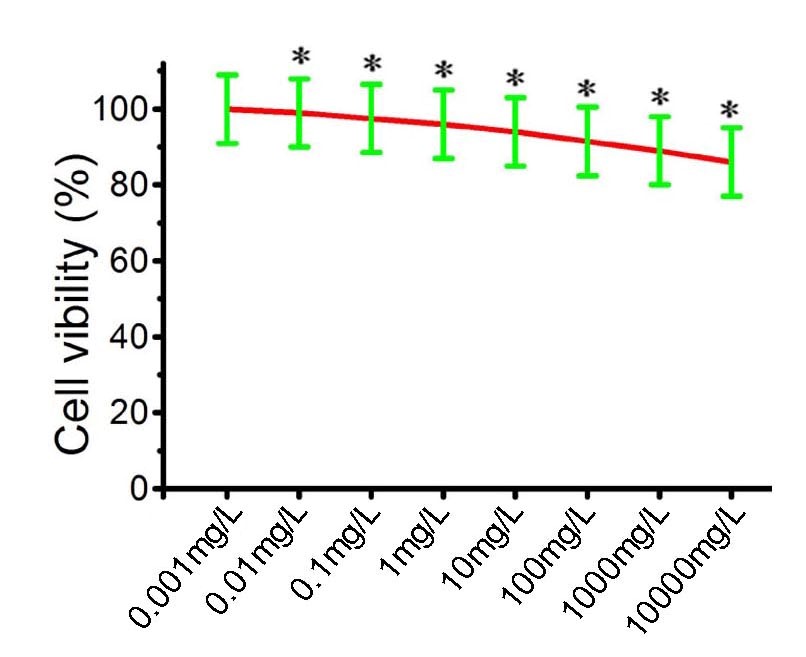


Figure S5.

MTT assay of HEK293 cell viability in the presence of Zinc NPs with different gradient concentrations.


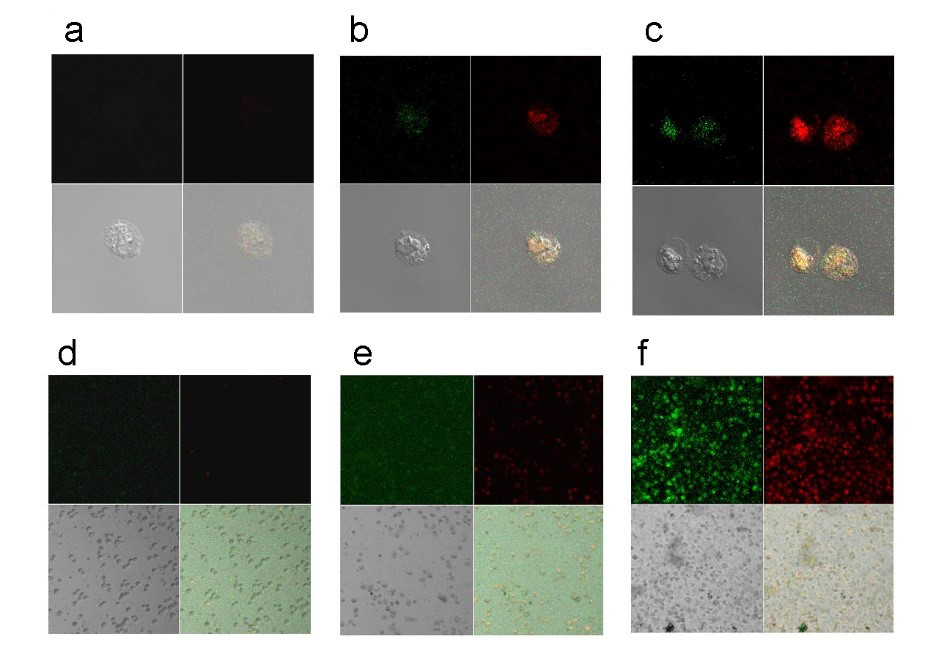


Figure S6.

Laser confocal fluorescence micrographs of primary leukemic cells from acute myeloid leukemia (AML) cells incubated with identical 10 mmol/L Zn2+ solutions. a, d: control; b, e: after 12 h incubation; c, f: after 48 h incubation. a, b, c were acquired at 400-fold magnification and d, e, f were acquired at 100-fold magnification.

## Table S1. The results of RNA-ChIP analysis of various protein variant types

| Spliced variant types | Genome mean intensitiesin treated cells | Genome mean intensitiesin control | | Fold in change (treated/control) | |
| --- | --- | --- | --- | --- | --- |
| Zinc finger protein |  | |  | |  |
| Zinc finger, AN1-type domain 2A | 1051.18 | | 362.76 | | 2.90 |
| Zinc finger protein 277 | 95.16 | | 25.93 | | 3.67 |
| Zinc finger protein 415 | 14.43 | | 11.96 | | 1.21 |
| Zinc finger protein 70 | 117.70 | | 98.11 | | 1.20 |
| Zinc finger protein  and BTB domain containing 42 | 185.85 | | 142.05 | | 1.31 |
| Zinc finger protein 323 | 71.90 | | 54.99 | | 1.31 |
|  |  | |  | |  |
| Glutathione s-transferase |  | |  | |  |
| Glutathione S-transferase | 21.28 | | 6.41 | | 3.32 |
| Glutathione disulfide  oxidoreductase activity | 14.32 | | 4.94 | | 2.89 |

## Note: The control corresponds to cells not exposed to 10 mM ZnCl_2_.
